# Modelling of adequate and safe vitamin D intake in Danish women using different fortification and supplementation scenarios to inform fortification policies

**DOI:** 10.1007/s00394-017-1586-9

**Published:** 2018-01-03

**Authors:** Ida M. Grønborg, Inge Tetens, Majken Ege, Tue Christensen, Elisabeth Wreford Andersen, Rikke Andersen

**Affiliations:** 1 National Food Institute, Technical University of Denmark, Lyngby, Denmark; 2 Department of Nutrition, Exercise and Sports, Copenhagen University, Frederiksberg, Denmark; 3 Institute of Applied Mathematics and Computer Science, Technical University of Denmark, Lyngby, Denmark

**Keywords:** Vitamin D, Fortification, Intake modelling, Danish women

## Abstract

**Purpose:**

Fortification of foods with vitamin D may be a population-based solution to low vitamin D intake. We performed modelling of vitamin D from diet, fortified foods and supplements in a population of Danish women 18–50 years, a risk group of vitamin D deficiency, to inform fortification policies on safe and adequate levels.

**Methods:**

Based on individual habitual dietary vitamin D intake of female participants from the Danish National Survey of Dietary Habits and Physical Activity (DANSDA) (*n* = 855), we performed graded intake modelling to predict the intake in six scenarios increasing the vitamin D intake from a habitual diet without fish to habitual diet including fish, fortified foods and supplements (40/80 µg). Four different foods were used as potential foods to fortify with vitamin D.

**Results:**

The vitamin D intake was below the Average Requirement (AR) of 7.5 µg/day for 88% of the assessed women. Safe levels of intake (< 100 µg/day) were observed after adding four different fortified foods (plain yoghurt, cheese, eggs and crisp-bread) contributing with a total of 20 µg/day and a vitamin D supplement of 40 µg/day to the habitual diet. Consumption of fish, fortified foods and a vitamin D supplement of 80 µg resulted in intakes above the Tolerable Upper Intake Level (UL) < 100 µg/day.

**Conclusions:**

In a Danish female population with a low vitamin D intake, low-dose fortification of different foods with vitamin D may be an effective and safe population-based approach.

## Introduction

Deficiency and insufficiency of vitamin D, defined as a serum 25-hydroxy vitamin D (25(OH)D) below 30 and 50 nmol/L, is a prevalent public health problem that applies for the Nordic countries mainly due to a 4- to 6-month-long winter period without sufficient sun exposure to initiate the cutaneous vitamin D production [1–3]. In Denmark, the prevalence of vitamin D status below 50 nmol/L has recently been estimated to be approximately 23% in a study of 3904 adults and an RCT with 420 adults; both studies were part of a recent Vitamin D Standardization Program (VDSP) publication [4]. A recent Danish survey showed that 11% of the adult population (*n* = 2625) had a vitamin D status below 25 nmol/L [5]. Sustained vitamin D deficiency (25(OH)D below 30 nmol/L) is known to be associated with risk of poor bone health, muscle pain and weakness [6–8]. Vitamin D status, measured as serum 25(OH)D, is affected by sun exposure, intake from the habitual diet and the consumption of vitamin D-containing supplements. The contribution from the habitual diet, however, is generally low. According to the most recently conducted Danish National Survey of Dietary Habits and Physical Activity (DANSDA) 2011–13 [9], the median dietary intake for women (18–75 years) in Denmark is 3.0 µg/day [10th and 90th percentiles (1.3; 9.0)], and for men the intake is 3.7 µg/day (1.8; 11.0). This current intake is considerably lower than the Average Requirement (AR) of 7.5 µg/day stated by the Nordic Nutrition Recommendations (NNR) [10]. In general, women have a lower dietary intake of vitamin D than men and are therefore at greater risk of deficiency [11].

The consumption of vitamin D supplements has proven to be effective in increasing vitamin D status, although this strategy is naturally only effective in those who consume the supplements and the risk of too high intakes is ever present [12]. Compared with other European countries, the consumption of dietary supplements in Denmark is high [13] and also the intake of vitamin D-containing supplements is high, and 57% of the women between 18 and 50 years have an intake of vitamin D supplements, self-reported in DANSDA [9]. Supplementation as a strategy holds risks of deficiency in non-consumers and toxicity in individuals with a ‘more is better’ approach, whereas food-based population strategies such as vitamin D fortification may be a potential future goal for countries like Denmark.

Vitamin D fortification has not yet been widely implemented and accepted in Denmark although voluntary fortification with vitamin D in selected products has been allowed since 2005 [14]. At present, only few products in categories such as fat spreads, sports drinks and lactose-free milk products are fortified with vitamin D. Low-dose fortification may be a strategy to increase the intake of those individuals in the lower end of the intake distribution range without increasing the risk of the upper end reaching toxic intake levels [12]. Previous studies from Denmark and Finland have shown that fortifying several foods with a low dose is a safer and more effective approach than fortifying a single food [15, 16]. Foods suited for vitamin D fortification have previously been identified to include milk and milk products, margarines, bread and juice because these foods are consumed by the majority of the assessed populations [15, 17, 18]. The chosen foods for fortification with vitamin D in our model were plain yoghurt, cheese, eggs and crisp-bread, all foods found in a habitual Danish diet and eaten by the majority of the population, but not yet available as fortified foods [9].

The objective of this study is to create an intake model focusing on Danish women at risk of vitamin D deficiency based on low-dose vitamin D-fortified foods using data from 18- to 50-year-old female participants from a representative national dietary survey (DANSDA). Dietary vitamin D intake from habitual diet, four different fortified foods and supplements were used in the model in order to inform fortification policies on safe and adequate levels in a Danish setting.

## Methods

**Fig. 1:**
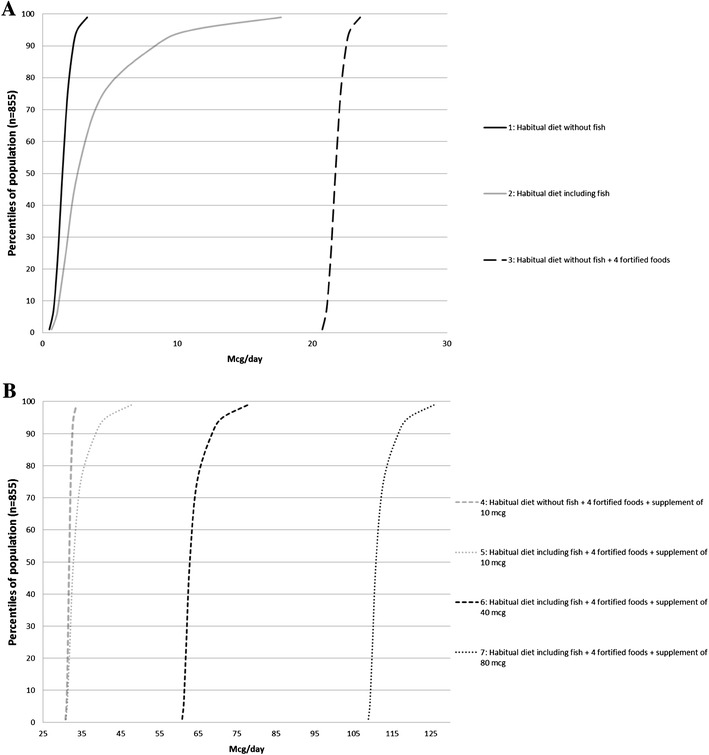
**a** Distribution of habitual vitamin D intake with and without fish, and addition of vitamin D-fortified foods contributing with 20 µg/day in a population of Danish women (*n* = 855). **b** Distribution of vitamin D intake in four scenarios adding foods fortified with vitamin D (20 µg/day) and different doses of vitamin D supplements in a population of Danish women (*n* = 855)

We performed a graded modelling of dietary vitamin D intake adding extra vitamin D from fortified foods and supplements to the habitual diet of 855 women. Individual intake data (dietary vitamin D) on 855 Danish (Caucasian) women aged 18–50 years were extracted from the Danish National Survey of Dietary Habits and Physical Activity (DANSDA) 2011–13 [9]. The 855 women had all completed seven consecutive days of individual dietary recordings. Using the 7-day dietary recordings combined with the Danish food databank [19], the median individual intake of dietary vitamin D was calculated, assuming that this was the habitual intake of the individuals. We calculated the percentiles and plotted the distribution of the population to illustrate the habitual intake and visualize the subsequent addition of fortified foods and vitamin D supplements in the model scenarios listed in Table 1 and shown in Fig. 1a, b.

**Table 1:** Description of the basic habitual diet and the six intake scenarios

Scenarios	Description of the scenarios
1	Vitamin D from habitual diet including fish
2	Vitamin D from habitual diet without fish
3	Scenario 2 + fortified foods^a^ (plain yoghurt, cheese, eggs and crisp-bread)
4	Scenario 2 + fortified foods^a^ (plain yoghurt, cheese, eggs and crisp-bread) + 10 µg/day supplement
5	Scenario 1 + fortified foods^a^ (plain yoghurt, cheese, eggs and crisp-bread) + 10 µg/day supplement
6	Scenario 1 + fortified foods^a^ (plain yoghurt, cheese, eggs and crisp-bread) + 40 µg/day supplement
7	Scenario 1 + fortified foods^a^ (plain yoghurt, cheese, eggs and crisp-bread) + 80 µg/day supplement

^a^The four fortified foods contribute with 20 µg/day vitamin D_3_ distributed in plain yoghurt, cheese, eggs and crisp-bread

The distribution of the habitual dietary intake of vitamin D excluding the intake from fish (scenario 1) calculated from the dietary recordings of the 855 individuals is shown in Fig. 1a. From this level, we added the intake of vitamin D from fish in order to get the habitual vitamin D intake of women that consume fish (Scenario 2) (Fig. 1a). We then added the four fortified foods (plain yoghurt, cheese, eggs and crisp-bread) contributing with approx. 20 µg/day (Scenario 3) (Fig. 1a). In the next scenario, a daily supplement of 10 µg was added (scenario 4) (Fig. 1b). Fish was then again added to the diet to explore whether some women eating fish and a daily supplement would be at risk of too high an intake (Scenario 5) (Fig. 1b). Finally, we added two different vitamin D supplements with doses of 40 and 80 µg/day as these supplements are sold and consumed in Denmark (Scenario 6–7) (Fig. 1b).

The choice of fortified foods and portion sizes (150 g plain yoghurt, 60 g cheese, 1 egg of 60 g egg and 10 g crisp-bread) were based on previous intervention studies and the Danish National Survey of Dietary Habits and Physical Activity (DANSDA) 2011–13 [9, 15] in order to fit the Danish dietary habits. In the modelling exercise, the assumption was that every person in the population would eat all of the fortified food portions assigned per day and consume 100% of the supplements given. This approach is a choice to ensure the safety of the entire population. The dose of vitamin D from the four fortified foods was approx. 20 µg/day which is the level recommended for persons at risk of osteoporosis in Denmark, and we found this level appropriate since our model was aimed at women with a very low intake of vitamin D and non-consumers of fish [20]. The daily dose of 20 µg/day of vitamin D_3_ was distributed in plain yoghurt, cheese, eggs and crisp-bread.

Different concentrations of vitamin D supplements were used in our model and these were chosen from a study of the Danes use of supplements [21], and the high doses represent doses that can legally be sold in Denmark as well as the internationally accepted Tolerable Upper Intake Level (UL) (< 100 µg/day).

The modelling was performed in Microsoft Excel, and vitamin D intake is represented as µg/day. The percentage of women below the Average Requirement (AR) of 7.5 µg/day was calculated based on the habitual intake of vitamin D obtained in a 7-day food diary from each of the 855 women (µg/day).

## Results

### Vitamin D intake scenarios

By use of individual habitual dietary vitamin D intake data from the 855 women in the nationally representative DANSDA survey, the initial habitual diet distribution and the six scenarios were created [9] (Fig. 1a, b). The specific vitamin D intakes at the percentiles 5–99 are listed in Table 2.

**Table 2:** Predictive modelling of total vitamin D intake distribution from habitual diet (HD), HD without fish, and five scenarios adding fortified foods and supplements in a population of Danish women (*n* = 855)

Scenarios (µg/day)	Percentiles					
	5	25	50	75	95	99
1 Vitamin D from HD including fish	1.0	1.7	2.6	4.5	11.1	17.7
**2** Vitamin D from HD without fish	0.7	1.1	1.5	1.9	2.6	3.3
**3** Scenario 2 + 4 fortified foods	21	21.4	21.7	22.1	22.8	23.6
**4** Scenario 2 + 4 fortified foods + 10 µg/day supplement	31	31.4	31.7	32.1	32.8	33.6
5 Scenario 1 + 4 fortified foods + 10 µg/day supplement	31.3	32	32.8	34.7	41.3	47.9
6 Scenario 1 + 4 fortified foods + 40 µg/day supplement	61.3	62	62.8	64.7	71.3	77.9
7 Scenario 1 + 4 fortified foods + 80 µg/day supplement	109.3	110	110.4	112.8	119.3	125.9

Fortified foods contribute with 20 µg/day vitamin D_3_ distributed in plain yoghurt, cheese, eggs and crisp-bread

All values in the table are represented in µg/day unless otherwise specified

The women of the DANSDA survey have a wide distribution of vitamin D intake ranging between 0.7 µg/day and 17.7 in the 1st and 99th percentiles (Fig. 1a; Table 2) (DANSDA 2011–13). The percentage of women below the AR of 7.5 µg/day given by the Nordic Nutrition Recommendations (NNR) was 88%, when looking at the habitual dietary intake (including fish), emphasizing the extensiveness of the problem affecting this population of women [8]. The intake of fish is responsible for the difference between the two first curves from the left (the black and the grey non-dashed curves) (Fig. 1a) and is a result of the large variation in the daily fish intakes and the fact that fish potentially contribute with a high concentration of vitamin D, depending on the type of fish. Among the women between 18 and 50 years, 57% reported consuming vitamin D-containing supplements and these women had a median intake of vitamin D of 9.5 µg/day from supplements. In our model, we follow a risk-averse approach and assume that 100% of the women consume the designated portions of fortified foods as well as supplements.

Scenarios 1 and 2 (habitual vitamin D intake not including fish and including fish) show the low intake levels between 0.7 and 4.5 µg/day at the 5th, 25th, 50th and 75th percentiles. Safe levels of intake below 100 µg/day are observed in Scenarios 3–5. Also in Scenario 6 the total vitamin D intake of the 99th percentile is considered safe and does not exceed 80 µg/day, which is below the tolerable Upper intake Level (UL) of 100 µg/day [20, 22]. Scenario 7 depicts a situation of a high vitamin D supplement intake (80 µg/day) and a diet that includes fortified foods, which results in all of the 855 women having a total intake above the UL of 100 µg/day. The maximum intake in scenario 7 was 132 µg/day.

## Discussion

The main findings of this paper are that in a setting of a low habitual dietary intake of vitamin D where 88% of the assessed female population had a habitual intake below the AR of 7.5 µg/day, the addition of four vitamin D-fortified foods, contributing with a total daily dose of 20 µg/day, was safe in a population of Danish women. Only those consuming an additional daily vitamin D supplement of 80 µg/day or more may be at risk of exceeding the UL of intake.

In Denmark, we observe an extremely low dietary intake of vitamin D and a relatively high intake of vitamin D from supplements, especially in certain population groups such as elderly women [9, 21]. This pattern is a result of the dietary preferences of the Danish people, current guidelines for intake of supplements and a lack of mandatory vitamin D fortification. Prior to implementing a national fortification programme, it is relevant to look at the possible effects and safety of such a programme in a model situation to predict the consequences on a population level.

In the present study, we chose to focus on Danish women in the modelling of vitamin D intake levels because women are known to have a lower intake of vitamin D than men, despite similar intake recommendations for the daily intake [9, 10]. Previous intervention and cohort studies have reported extremely low vitamin D status (defined as 25(OH)D < 12 nmol/L) in women [23, 24]. Some of the reasons for women having a lower intake may be due to a lower intake of calories and meat compared to men [9].

After reviewing the literature for foods well suited for vitamin D fortification, we identified the most commonly fortified foods as being milk and milk products, margarines, bread and juice [15, 17, 18]. Eggs appeared as an interesting novel food and a good bio-fortified (vitamin D added to the animal diet) source of vitamin D [25]. We chose the four foods based on relevancy in a Danish setting. The fortified foods were yoghurt, cheese, eggs and crisp-bread, all foods included in a habitual Danish diet and eaten by the majority of the population [9]. Low-dose fortification was chosen to create a realistic model for a Danish fortification situation. The use of several foods in the fortification model and lower concentrations of vitamin D in each food makes it more safe and efficient, whereas using a single food such as milk may be problematic because non-consumers skew the intake across a population [16, 26].

In the modelling, each individual in the population (DANSDA) was assumed to eat the same daily portion of the four fortified foods. This assumption makes the curve shift equally in every percentile as shown in Fig. 1a, b. Adding a vitamin D supplement of 10 µg to the diet again shifts the entire population under the assumption that everyone has an equal intake, except for the individual habitual dietary intake. These assumptions ensure the safety of the fortification levels when each individual has a daily consumption of the foods of plain yoghurt (150 g), cheese (60 g), one egg (60 g) and one crisp-bread (10 g). In a real-life situation, we will inevitably see varying consumption patterns of the fortified foods, meaning that not all will eat the proposed amount used in our model. However, we chose to perform the modelling with a risk-averse approach and the above assumptions to ensure the safety of the entire population in case of the introduction of a national fortification scheme in Denmark. In this way, we have a margin of safety, which in our view is ideal when dealing with a fat-soluble vitamin such as vitamin D.

The NNR has stated an AR of vitamin D for the age group of 7.5 µg/day and The Danish National Health Authorities recommend a vitamin D intake of 10 µg/day for children and adults and 20 µg/day for elderly (> 70 y) and risk groups of osteoporosis (Danish National Health Authority 2010). Therefore, our goal was to increase the intake of the Danish women to 20 µg/day from fortified foods, to ensure that this risk group of a low vitamin D intake will have their vitamin D status lifted and maintained throughout the year. The scenarios including extra vitamin D supplements simulate the real-life situation in which the intake of supplements in Denmark is high [5, 21, 27].

### Strengths and limitations

A clear strength of this paper was the inclusion of individual consumption data from a nationally representative survey which provides results applicable to a Danish setting. These data allow for the use of these results in a Danish setting by policy makers and public health agencies. We did not assess all levels of supplementation, only the chosen three levels being 10, 40 and 80 µg/day as well as the UL level of 100 µg/day.

## Conclusion

The current vitamin D intake was below the AR of 7.5 µg/day in 88% of the 855 Danish women assessed in this paper. By performing modelling of vitamin D fortification in the population of Danish women 18–50 years, we showed that adequate and safe levels of intake were present in all women consuming the fortified foods and a daily supplement of vitamin D as high as 40 µg/day. When consuming a daily vitamin D supplement of 80 µg/day or more, all of the women were at risk of reaching the UL of intake (100 µg/day).
